# Complications after percutaneous device closure of atrial septal defects in children: Prevalence, outcomes and associated factors

**DOI:** 10.1016/j.ijcchd.2026.100672

**Published:** 2026-03-27

**Authors:** Michael N. Gritti, Olivia Carere, Brian W. McCrindle, Rajiv R. Chaturvedi, Lee N. Benson

**Affiliations:** a Temerty Faculty of Medicine, University of Toronto, Toronto, Ontario, Canada; b Labatt Family Heart Centre, Hospital for Sick Children, Toronto, ON, Canada

**Keywords:** Pediatrics, Congenital heart defect, Atrial septal defect, Transcatheter closure, Long-term outcomes, Complications

## Abstract

**Background:**

Atrial septal defects (ASDs) represent 10% of congenital heart disease. Transcatheter closure is the standard of care for anatomically suitable defects, but pediatric long-term outcomes remain underreported. This study examined the prevalence, outcomes, and risk factors for complications following ASD device closure in children.

**Methods:**

We retrospectively reviewed children <18 years undergoing transcatheter ASD closure at a single tertiary center (1989–2016). Demographic, procedural, and follow-up data were analyzed. Kaplan-Meier methods assessed freedom from major complications and associations with device type, imaging technique, and concomitant congenital heart disease (CHD).

**Results:**

A total of 971 children (mean age 8.2 ± 4.2 years, 62% female) underwent closure, with median follow-up 2.2 years (maximum 20.7). Concomitant CHD was present in 33%. Major complications occurred in 1.6%, including device embolization (0.6%), erosion (0.4%), and urgent surgery (0.6%). Rates were highest with early-era devices (8%). Kaplan-Meier analysis showed freedom from major complications of 98.1% at 5 years and 97.6% at 10 years. No significant differences were observed by imaging modality (ICE vs. TEE) or presence of concomitant CHD.

**Conclusions:**

In this large pediatric cohort, transcatheter ASD closure was associated with low rates of major complications and excellent long-term outcomes. Neither concomitant CHD nor imaging technique significantly affected complication risk. These findings reinforce the long-term safety of ASD device closure in children.

## Highlights

• A large pediatric cohort of atrial septal defect device closures with medium to long term follow-up.
• Major complication rate was low at 1.7% with excellent 10 year outcomes.
• Major complication risk did not differ by procedural imaging modality or concomitant congenital heart disease.
• Multiple defects were safely closed without increased complication risk.
• Findings reinforce sustained safety of ASD device closure in children.

## Abbreviations

**ASD**: Atrial Septal Defect
**AV**: Atrioventricular
**CHD**: Congenital Heart Defect
**EP**: Electrophysiology
**ICE**: Intracardiac Echocardiography
**mGy**: Milligray
**OR**: Odds ratio
**PFO**: Patent Foramen Ovale
**TEE**: Transesophageal Echocardiography
**TTE**: Transthoracic Echocardiography

## 1 Introduction

Atrial septal defects (ASD) are among the most common congenital heart lesions, accounting for approximately 10% of all congenital heart defects (CHD) [1]. While often asymptomatic in early life, a prolonged left to right shunt and right heart volume overload can result in right heart dysfunction and pulmonary hypertension [2]. Transcatheter closure is considered the standard of care for anatomically suitable defects as an alternative to surgical repair [3], and is associated with a high procedural success and outcomes comparable to surgery [[4], [5], [6]]. Despite literature reporting the long-term safety of transcatheter techniques in adults [7,8], there is a paucity of data regarding outcomes in childhood [1,9]. Previous studies focusing on children have largely reported acute procedural outcomes, leaving a gap in our understanding of the medium to long-term clinical outcomes [[10], [11], [12], [13]]. This study assessed the prevalence, outcomes and associated factors associated with complications for children who underwent transcatheter ASD device closure at a large tertiary care center.

## 2 Methods

### 2.1 Population and data collection

Institutional databases were searched to identify all children who underwent transcatheter ASD device closure at the Hospital for Sick Children between August 1989 and November 2016. Eligible children were <18 years of age at the time of the procedure, regardless of associated CHD. Exclusion criteria involved cases where data were incomplete. Medical records, including operative and catheterization reports, were reviewed to abstract demographic and procedural data. Collected variables included age, gender, primary diagnosis, concomitant CHDs, family history of an ASD, defect morphology (size and number of defects), intraprocedural echographic imaging technique and device implanted as well as fluoroscopy time and radiation exposure.

Clinical complications during and after the procedure, were categorized as either major or minor. Major complications were defined as any event that required escalation in management or had long term sequelae. This included device embolization, erosion, urgent surgical intervention, and blood transfusion. Minor complications were defined as unanticipated events that had no sequelae and did not require a change in management, including transient arrhythmias and vascular site issues.

### 2.2 Statistical analysis

Descriptive statistics were used to summarize patient demographics and procedural characteristics. Continuous variables were reported as either mean ± standard deviation or median with interquartile range (values at the 25th and 75th percentiles). Categorical variables were expressed as frequencies and percentages. Overall prevalence of major, minor, and individual complications was calculated across the full cohort. Kaplan-Meier survival analyses were used to estimate freedom from major complications over time, with separate analyses for specific complications including device embolization, device erosion, and urgent surgeries. Associations between complication rates and clinical factors including the presence of concomitant CHD, occluder type, imaging modality (transesophageal echocardiography [TEE] *vs.* intracardiac echocardiography [ICE]) and number of ASDs were assessed using Fisher's exact test for categorical variables. For binary subgroup comparisons, using 2x2 contingency tables, odds ratios (OR) with 95% confidence intervals (CI) were reported. For multi-group comparisons (such as device type), pairwise comparisons were conducted. To assess temporal trends across procedural eras, logistic regression models were used, treating procedural era as an ordinal variable to evaluate for linear changes in major and minor complication rates over time. All analyses were performed using R version 4.0.3. Statistical significance was set at p < 0.05. The study was approved by the Research Ethics Board at The Hospital for Sick Children with a waiver of informed consent due to its retrospective design.

## 3 Results

### 3.1 Study cohort and demographic information

During the study period, 971 children underwent transcatheter ASD device closure. The mean age was 8.2 ± 4.2 years and weight 30.1 ± 17.0 kg. The cohort was 62% (n = 599) female. Six hundred and fifty-two children (67%) had an isolated secundum ASD, while 319 (33%) had an additional cardiac lesion. A positive family history of an ASD was present in 41 children (9% of 484 children with available data). Genetic anomalies were reported in 71 children (7%), including 35 with trisomy 21 (4%). The median follow-up duration was 815 days (2.2 years, IQR 58-2395 days), with a maximum of 20.7 years. Baseline characteristics are summarized in Table 1.

**Table 1:** Demographic data on cohort of patients who had a transcatheter ASD device insertion at SickKids between 1989 and 2016 (n = 971).

Variable	Frequency (%); Mean (SD)
**Patients**	971
**Age** (years)	8.2 (4.2)
**Gender** (male)	372 patients (38%)
Gender of patients with CHD (male)	114 patients (36%)
Gender of patients without CHD (male)	258 patients (40%)
**Weight** (kg)	30.1 (17.0)
**Body surface area** (m^2^)	1.01 (0.4)
**Other congenital heart defects**	319 patients (33%)
**Family History of ASD**	41 patients (9%)
**Genetic Anomalies**	71 patients (7%)
Trisomy 21	35 patients (4%)
**History of Prematurity**	153 (18%)

### 3.2 Baseline anatomical and physiologic characteristics

There were 822 children with a single secundum ASD (85%), with 140 (15%) having multiple defects (Table 2). For single defects the mean size was 14.0 ± 4.8 mm (from intraprocedural echocardiogram) and 17.1 ± 5.1 mm from balloon sizing. For those with multiple defects, the largest defect measured 12.1 ± 4.4 mm (by echocardiogram) and 15.7 ± 4.5 mm by balloon sizing. The mean implanted device size was 21.1 ± 6.9 mm (median 20 mm (IQR 4-40)). Mean estimated right ventricle systolic pressure from baseline echocardiogram was 30.4 ± 9.9 mmHg. At catheterization the mean pulmonary artery pressure was 17.5 ± 5.6 mmHg and a mean right ventricular systolic pressure 32.5 ± 12.1 mmHg. Defect characteristics are summarized in Table 2.

**Table 2:** ASD characteristics and procedural data of n = 971 patients who underwent transcatheter ASD device insertion.

Variable	Frequency (%); Mean (SD)
**Number of ASD**	
1	822 patients (86%)
2	123 patients (13%)
3	15 patients (2%)
4+	2 patients (0.2%)
**ASD Size (mm) - Echo**	13.7 (4.8)
**ASD Size (mm) - Balloon**	16.8 (5.1)
**Device Size (mm)**	21.1 (6.9)
**Echo Technique in Lab**	
TEE/TTE	684 patients (77%)
ICE	203 patients (23%)
**Number of Devices Required**	
1	943 patients (97%)
2	26 patients (3%)
3	2 patients (1%)
**Device Type**	
Amplatzer Septal Occluder	791 patients (81%)
Gore Helex Septal Occluder	32 patients (3%)
CardioSEAL	62 patients (6%)
Clamshell	53 patients (5%)
Amplatzer PFO	15 patients (2%)
BioSTAR	13 patients (1%)
Amplatzer Cribiform Occluder	4 patients (0.9%)
Rashkind	1 patient (0.1%)
**Radiation Exposure (mGy)**	103.8 (171.1)
**Fluoroscopy Time (minutes)**	10.4 (10.6)

### 3.3 Procedural characteristics

**Fig. 1:**
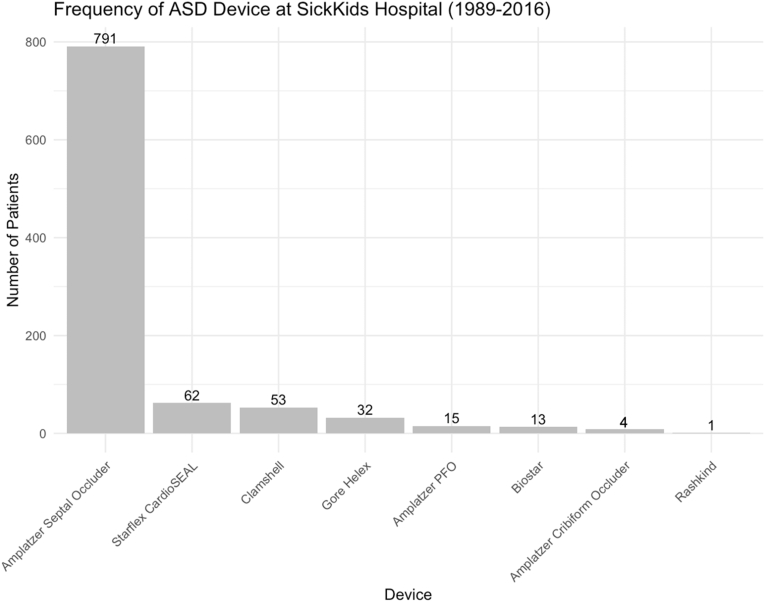
Frequency of ASD Device Usage at SickKids Hospital (1989–2016). The number of patients treated with each device type is shown.

Transesophageal echocardiography (TEE) was used during 684 procedures (77%), while intracardiac echocardiography (ICE) guidance was used in 203 (23%). A single device was used in 943 procedures (97%), while two devices were implanted in 26 procedures (3%) and 2 procedures (<1%) required three devices. The median fluoroscopy time was 7.8 min (IQR 5–13). Median fluoroscopy time for procedures using ICE imaging was 7.6 min (IQR 5–12), compared to 8.0 min (IQR 5–14) for procedures using TEE. The median radiation exposure was 60.0 mGy (IQR 32–122). Devices used for included the Amplatzer Septal Occluder (ASO) (*Abbott*; n = 791, 81%), CardioSEAL (*NMT Medical*; n = 62, 6%), Clamshell Occluder (*Clamshell Inc*.; n = 53, 5%), Gore Helex Septal Occluder (GSO) (*W. L. Gore & Associates*; n = 32, 3%), Amplatzer PFO Occluder (*Abbott*; n = 15, 2%), BioSTAR (*NMT Medical*; n = 13, 1%), Amplatzer Cribiform Occluder (*Abbott*; n = 4, 0.9%), and Rashkind Occluder (n = 1, 0.1%) devices (Fig. 1). Procedural characteristics are summarized in Table 2.

### 3.4 Prevalence and outcomes of complications

**Fig. 2:**
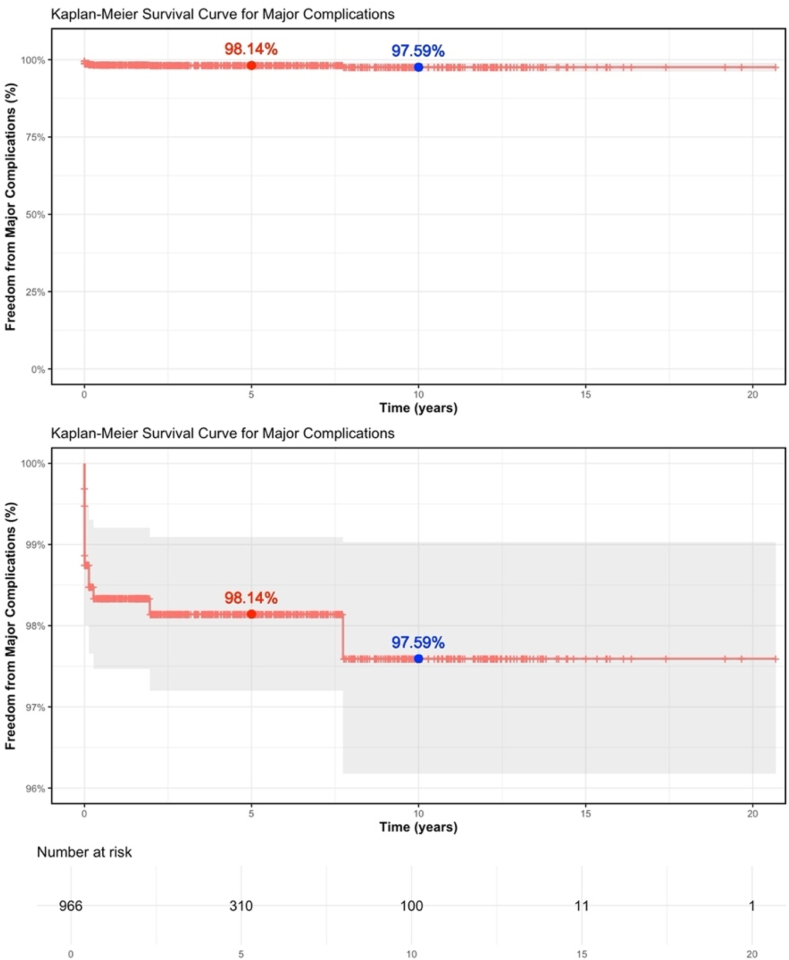
Kaplan-Meier survival curve for freedom from major complications following transcatheter ASD closure.

**Fig. 3:**
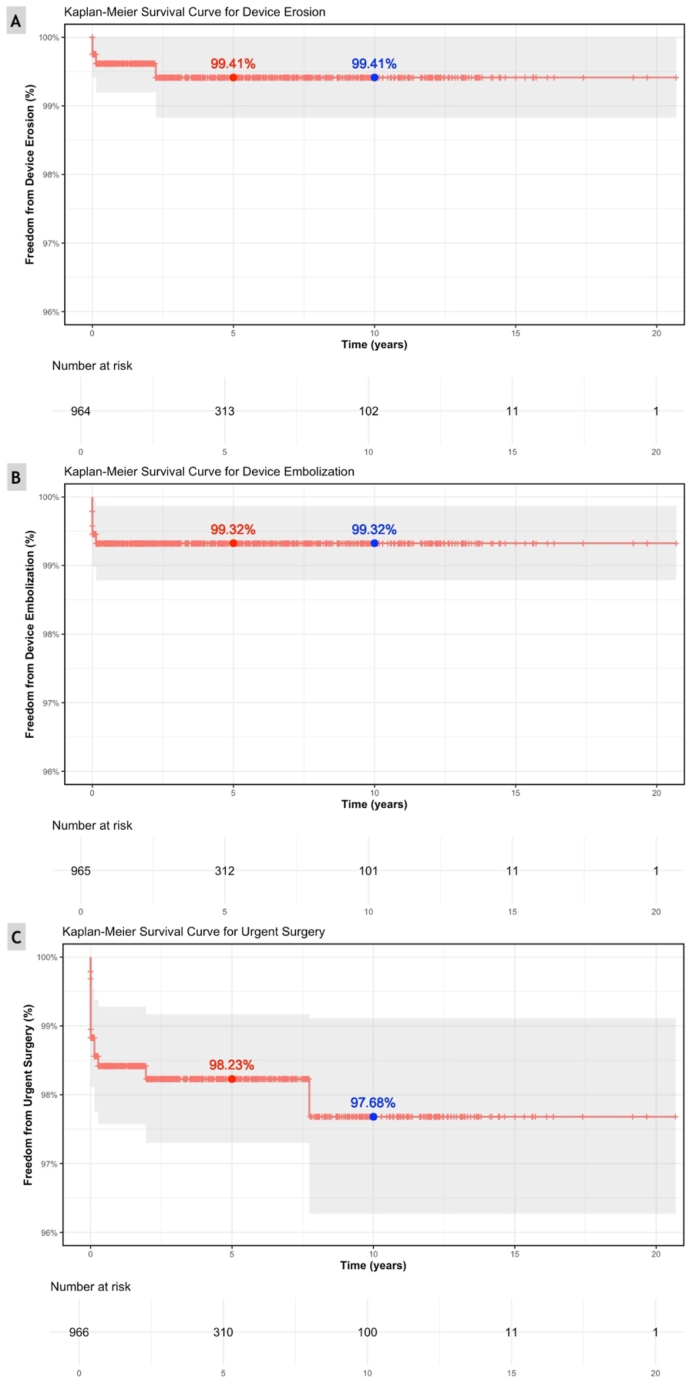
Kaplan-Meier survival curves for specific major complications following transcatheter ASD closure. (A) Freedom from device erosion. (B) Freedom from device embolization. (C) Freedom from urgent surgery.

*Major complications*: During the study period, 17 children (1.7%) had major complications (Table 3). Among these, device embolization was observed in 6 (0.6%), with 5 events presenting within the first 2 days after implantation, and 1 event occurring 49 days after implantation. Urgent surgical retrieval was required in 4 children (0.4%), while 2 were managed with catheter-based snare retrieval and device re-implantation. Device erosion occurred in 4 children (0.4%), all within 49 days after implantation. Presentations included 1 case each with complete heart block or a pericardial effusion and 2 cases of high grade second-degree atrioventricular block. All episodes of erosion were managed with urgent surgical device removal and surgical ASD closure. Electrophysiologic complications requiring urgent surgical intervention occurred in 4 patients (0.4%), 2 patients from the erosion group and 2 additional patients who developed complete heart block within 48 hours of the procedure. An additional 4 children (0.4%) required urgent surgical intervention for other indications. A blood transfusion was required during 1 procedure (0.1%). There were no deaths in this cohort. The unadjusted Kaplan-Meier freedom from a major complication (Fig. 2) for the cohort at 5 years was 98.1%, slightly decreasing to 97.6% at 10 years. The survival probability for freedom from device erosion at 5 and 10 years was 99.4% (Fig. 3a). For embolization, the freedom from embolization probability was 99.3% at both 5 and 10 years (Fig. 3b). The Kaplan-Meier curve for urgent surgery shows a slight decrease in freedom from surgery probability to 98.2% at 5 years and 97.7% at 10 years (Fig. 3c).

**Table 3:** Frequency and percentage of major and minor complications following transcatheter ASD device closure (N = 971).

Variable	Frequency (%)
**Major**	17 patients (1.7%)
Death	0 patients (0.0%)
Urgent Surgery	15 patients (1.6%)
Device Embolization	6 patients (0.6%)
Device Erosion	4 patients (0.4%)
Urgent Surgery due to EP issue	4 patients (0.4%)
Blood transfusion	1 patient (0.0%)
**Minor**	84 patients (8.5%)
Transient Arrythmia	62 patients (6.4%)
Vascular site	24 patients (2.4%)

*Minor Complications*: Minor complications were identified in 86 children (9%) including transient arrhythmias 62 patients (6%), and vascular site complications in 24 children (2%) (Table 3).

### 3.5 Factors associated with complications

*Complications associated with concomitant CHD*: Major complications occurred in 9 children with CHD (2.8%) and 8 patients with an isolated ASD (1.2%). There was no statistically significant difference between groups for major (OR 2.4, 95% CI 0.8–7.1, p = 0.1) or minor complications (OR 1.5, 95% CI 0.9–2.3, p = 0.1). There was more radiation exposure in those with concomitant CHD *vs*. those with just an isolated secundum ASD (117.1 ± 186.5 mGy vs 98.4 ± 164.9 mGy).

*Complications associated with defect number*: Among the 822 children with a single defect, 15 (2%) experienced major complications. In the 140 children with multiple ASDs, no child experienced a major complication. No statistically significant difference was observed in major complications compared to isolated defects (OR 0, 95% CI 0.0–1.6, p = 0.1).

*Complications associated with imaging technique*: TEE was utilized in 684 children (77%), with 9 (1%) experiencing a major complication. ICE was used in 203 children (23%), with 6 (3%) experiencing a major complication. No statistically significant differences were found between TEE and ICE for major complications (OR 2.3, 95% CI 0.7–7.3, p = 0.1).

*Complications associated with occlude type*: The ASO™ was the most frequently implanted device (n = 791), with 11 children (1%) experiencing a major complication. The Clamshell™ septal occluder and Amplatzer PFO™ device had the highest prevalence of major complications, with 4 events among 53 Clamshell septal occluder recipients (8%) and 1 event amongst 15 Amplatzer PFO™ Occluder recipients (8%). No major complications were observed among children who received the Gore Helex ™ (n = 32), CardioSEAL ™ (n = 62), Rashkind (n = 1), BioSTAR ™ (n = 13), or Amplatzer Cribriform Occluder ™ (n = 4). The Clamshell ™ device was associated with a significantly higher rate of major complications compared to the ASO ™ (8% *vs*. 1%, p = 0.01).

**Fig. 4:**
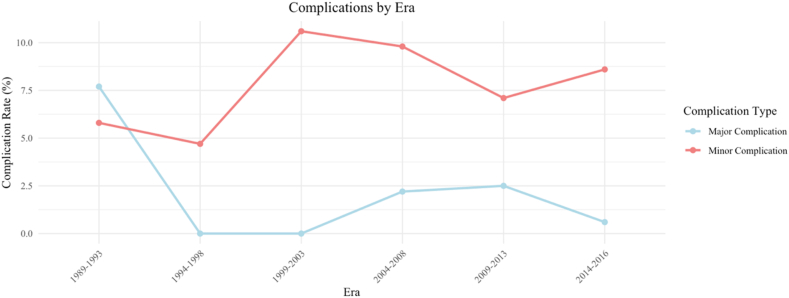
Complication prevalences over different procedural eras.

*Complications related to procedural era*: Complications stratified by procedural era (Fig. 4) showed a decline in major complications, from 8% (4 of 50 procedures) between 1989 and 1993 to 1% (1 of 97 procedures) in 2014–2016. A logistic regression model testing for a linear trend across eras showed no significant association between procedural era and major complication risk (OR 0.82, 95% CI 0.58–1.17, p = 0.25).

## 4 Discussion

This large single-center study of 971 children undergoing transcatheter ASD closure provides robust evidence of the safety of this intervention. The overall prevalence of major complications was low (1.7%), consistent with previous reports ranging from 1.0% to 1.8% [1,5,11,12,14], reinforcing a favorable safety profile. The most common minor complication was a transient arrythmias (6%), comparable to rates in other large pediatric cohorts of transcatheter ASD closure [9,15].

### 4.1 Device embolization

Amongst specific complications, device embolization was the most common (0.6%), consistent with reported prevalences between 0.2% and 1.7% [1,16]. Most embolizations were detected within 48 hours of implantation, supporting the need for close early observation. At our centre, we perform an echocardiogram 24 hours after the implantation, which would reasonably capture the majority of these events. One episode of embolization occurred beyond the initial procedural admission (1.5 months after the procedure), highlighting the importance of close follow-up within the first year.

### 4.2 Device erosion

Device erosion remains a serious complication of ASD device implantation, particularly seen after deployment of the ASO™^16^. Although newer, softer devices are being developed to reduce this risk, this remains a safety concern [17]. In our study, device erosion occurred in 0.4% of implants, aligning with previous reports of 0.04%–0.9% [5,8]. Amin et al. reported an erosion prevalence of 0.1% in a paediatric cohorts, with higher risk for procedures involving oversized devices or the presence of deficient aortic and/or superior rims [18]. In our study, most device erosions presented within 72 hours after implantation, while one presented 1.5 months after implantation. This underscores the need for long-term follow-up.

### 4.3 Electrophysiologic complications

EP complications requiring urgent surgical intervention, independent of erosion, occurred in 0.2% of the cohort involving high-grade conduction disturbances intra-procedurally. Urgent surgical removal was required when arrhythmias persisted post-procedure. These findings align with reports that while transient AV conduction delays often resolve, higher-grade heart block warrants urgent attention [19]. Jalal et al. similarly reported 2 episodes of intra-procedural complete AV block necessitating urgent surgical ASD closure [5]. These findings underscore the importance of close early follow-up for those who develop significant conduction disturbances during catheterization and the need for electrocardiographic monitoring during follow up.

### 4.4 Time to event analysis

Kaplan-Meier analysis demonstrated excellent outcomes over time, with a mean follow-up of 3.9 years (median 2.2 years [IQR 0.2–6.6 years], max 20.7 years). This is comparable to the 3.5-year mean follow-up in Jalal et al.’s cohort and the pooled mean of 2.7 years reported in Kashyap et al.’s review [1,5]. The probability of freedom from major complications was 98.1% at 5 years and 97.6% at 10 years. While these findings demonstrate sustained safety, they also reveal that the risk, though minimal, does not plateau entirely**.** Notably, two major urgent surgical interventions occurred 2- and 8-years post-implantation, supporting the need for extended, follow-up in pediatric patients.

### 4.5 minor complications

The most common minor complication was transient arrythmias (6%), comparable to rates in other large pediatric cohorts of transcatheter ASD closure^9,199^.

### 4.6 Concomitant CHD

Our study uniquely included children with concomitant CHD, a group often excluded from prior retrospective analyses [1]. The presence of concomitant CHD was not associated with a significantly increased prevalence of major complications. This supports clinical practice where ASD closure is offered in anatomically suitable children with other CHD lesions without added procedural risk [20].

### 4.7 Multiple secundum ASDs

Children with multiple defects did not experience a higher prevalence of major complications compared to those with a single defect. While prior studies support the feasibility of closing multiple ASDs, few stratify complications by defect number [21]. This findings suggests that device closure remains a safe option in the setting of fenestrated anatomy, provided appropriate device selection and procedural planning.

### 4.8 Imaging technique

TEE was the predominant imaging technique, with a low major complication prevalence of 1%. Although ICE was associated with a higher prevalence of major complications (3%) compared to TEE, this difference was not statistically significant. Prior paediatric studies have shown ICE to be a safe alternative to TEE, with advantages such as avoidance of general anesthesia and improved visualization of septal anatomy in select cases [[22], [23], [24], [25]]. Our findings support the safety of both imaging modalities when selected appropriately with operator competence.

### 4.9 Device type

The ASO™ demonstrated the lowest prevalence of major complications (1%), aligning with literature supporting its favorable risk profile compared to other devices [[24], [25], [26], [27]]. The Clamshell™ septal occluder device had a significantly higher prevalence of major complications (8%), likely due to its rigid structural design [28].

### 4.10 Era trends

Major complication rates declined over time, with the earliest era (1989–1993) showing the highest prevalence (8%), likely reflecting both the learning curve and the use of high-risk devices like the Clamshell device. Despite overall improvements, linear trends across eras were not statistically significant, suggesting that advances have likely plateaued.

## 5 Limitations

Our study is limited by its retrospective design and the absence of detailed referral indications, restricting assessment of procedural appropriateness. The data span over two decades, during which device technology and techniques evolved, affects generalizability. Follow-up duration varied, and some patients received care outside the tertiary center, which may have limited complete capture of late events.

## 6 Conclusions

Transcatheter closure of secundum ASDs is a safe procedure in paediatric patients, including those with concomitant CHD or multiple defects. The procedure can be routinely performed under TEE or ICE guidance. Our findings highlight that complications can arise beyond the initial procedural admission, emphasizing the importance of sustained follow-up beyond the immediate procedural period. Future work should explore strategies to optimize follow-up protocols and further delineate risk factors in anatomically complex cases.

## CRediT authorship contribution statement

**Michael N. Gritti:** Conceptualization, Data curation, Formal analysis, Methodology, Project administration, Resources, Validation, Writing – original draft, Writing – review & editing. **Olivia Carere:** Formal analysis, Methodology, Project administration, Writing – original draft. **Brian W. McCrindle:** Data curation, Formal analysis, Investigation, Methodology, Supervision, Writing – review & editing. **Rajiv R. Chaturvedi:** Conceptualization, Methodology. **Lee N. Benson:** Conceptualization, Formal analysis, Funding acquisition, Methodology, Supervision, Writing – review & editing.

## Data availability statement

The data that support the findings of this study are available from the corresponding author upon reasonable request.

## Ethics approval

Approved by the Research Ethics Board at The Hospital for Sick Children.

## Source of funding

None.

## Declaration of competing interest

The authors declare that they have no known financial, personal, or professional relationships that could have appeared to influence the work reported in this paper.
