# Supplementary material for: Complications after percutaneous device closure of atrial septal defects in children: Prevalence, outcomes and associated factors
Source: Int J Cardiol Congenit Heart Dis. 2026 Mar 27;24:100672. doi: 10.1016/j.ijcchd.2026.100672 (PMC13091151; doi:10.1016/j.ijcchd.2026.100672)
Supplement: Multimedia component 1 [file mmc1.docx]

| **Year** | **Major Complication** | **Age at Closure, years** | **Weight, kg** | **Comorbidity** | **Time to Compli-cation** | **Case Description** | **Device (type, size)** | **Imaging Tech-nique** |
| --- | --- | --- | --- | --- | --- | --- | --- | --- |
| 1989 | Urgent Surgery | 6.3 | 21.5 | Family History of ASD | 0 days | Complication: Clamshell device was deployed but not released due to a deficient superior septum observed on TEE. Attempts to remove the device resulted in it becoming lodged in the inferior vena cava.  Management: Urgent surgery was performed the same day to successfully remove the device and close the ASD. | Clam-shell 33mm | TEE |
| 1989 | Embolism & Urgent Surgery | 1.0 | 68 | CHD & Trisomy 21 | 1.5 months | Complication: 49 days PO, the device embolized and dislodged into the right ventricular outflow tract.  Management: Urgent surgical device retrieval & ASD closure. | Clam-shell 23mm | TEE |
| 1990 | Embolism & Urgent Surgery | 3.6 | 15.4 | Cardiac disease (critical PS, s/p valvotomy and right BT shunt with ligation) | 1 day | Complication: Pt with deficient inferior rim received a 40mm Clamshell device. Device embolized into the left atrium within 24 hrs, as confirmed by CXR (validated by TTE and TEE at 48 hours), positioned above the mitral valve, w/o causing inflow obstruction.  Management: Urgent surgical device retrieval & ASD closure. | Clam- shell 40mm | TEE |
| 1990 | Embolism & Urgent Surgery | 16.4 | 66 | Pulmonary Stenosis | 0 days | Complication: In the recovery room, the pt developed frequent PVCs. Echo revealed device embolization to the right ventricle.  Management: Due to ventricular irritability, the pt was treated with lidocaine and underwent urgent surgical removal of the device with ASD closure on the same day. | Clam-shell 40mm | TEE |
| 2004 | Erosion & Urgent Surgery | 14.6 | 64.7 | Left Atrial Isomerism | 49 days | Complication: Pt presented 49 days post operatively with chest pain and fainting, and was found to have a moderate pericardial effusion, primarily around the right atrium and right ventricle. The effusion was related to device erosion, as evidenced by bloody fluid upon drainage.  Management: Urgent surgical device removal & ASD closure. | ASO 18mm | ICE |
| 2004 | Urgent Surgery | 7.9 | 36.3 | Undiagnosed homozygous MTHFR mutation with associated clotting disorder | 7.7 years | Complication: 15.6-year-old pt experienced a transient ischemic attack (TIA) due to thromboembolism adjacent to the left side of the implanted ASD device 7.7 years after closure.  Management: Urgent surgical device retrieval & ASD closure following the TIA to mitigate further thromboembolic risk. | ASO 17mm | TEE |
| 2005 | Heart Block & Urgent Surgery | 5.2 | 19.3 | Isolated sec ASD | 2 days | Complication: Pt developed complete heart block during procedure, which improved before the device was released. By the evening, pt experienced intermittent junctional bradycardia and first-degree heart block.  Management: Device was surgically removed, and the ASD was closed. Post-surgery, junctional rhythm resolved, but 1st-degree heart block persisted. | ASO 24mm | TEE |
| 2007 | Heart Block & Urgent Surgery | 5.3 | 16.3 | CHD, h/o SVT and atrial flutter | 2 days | Complication: The child exhibited intermittent type 2 heart block during the catheterization, which was initially considered unrelated to the ASD device due to a history of SVT and atrial flutter.  Management: The following day, the patient developed intermittent complete heart block, leading to the decision to surgically remove the device. | ASO 24mm | TEE |
| 2008 | Embolism & Urgent Surgery | 7.9 | 23.4 | Isolated sec ASD | 2 days | Complication: The device embolized into the left atrium the day following implantation.  Management: Urgent surgical device retrieval & ASD closure. | ASO 24mm | TEE |
| 2009 | Erosion & Urgent Surgery | 5.6 | 15.9 | Isolated sec ASD | 3 days | Complication: 2 days post operatively, the pt was found to have a compromised inferior rim leading to a device erosion.  Management: Urgent surgical device removal & ASD closure. | ASO 28mm | TEE |
| 2009 | Urgent Surgery | 3.4 | 14.7 | CHD (Ebstein's anomaly), s/p VSD device closure | 1.9 years | Complication: 5 year old pt with a small right ventricle and tricuspid regurgitation had a significant residual VSD leak.  Management: Both ASD and VSD devices were surgically removed, and the defects were closed. | ASO 22mm | ICE |
| 2011 | Erosion, Heart Block & Urgent Surgery | 8.2 | 20.8 | Isolated sec ASD | 2 days | Complication: During device implantation, the pt developed 1st degree AV block that rapidly progressed to complete heart block due to device erosion impacting the AV node.  Management: Urgent surgical device removal & ASD closure. Post-procedurally, the pt returned to normal sinus rhythm w/o further complications. | ASO 28 mm | ICE |
| 2012 | Urgent Surgery | 12.1 | 41.1 | CHD (TOF, pulmonary stenosis) | 3 months | Complication: Pt originally presented with TIA and underwent device closure for a PFO. On FU, residual leak was identified and found to be due to an undocumented left SVC to an unroofed coronary sinus into the left atrium.  Management: Urgent surgical intervention. | Ampla-tazer PFO 25mm | ICE |
| 2012 | Embolism | 14.9 | 62.3 | CHD (TOF, pulmonary stenosis), sec ASD | 0 days | Complication: Device embolized to the aorta within 24 hrs.  Management: The embolized device was successfully retrieved via catheter using a 35mm gooseneck snare, and a new device was implanted on the 17th through a repeat catheterization. | ASO 19mm | TEE |
| 2012 | Erosion, Heart Block & Urgent Surgery | 4.5 | 14.4 | Isolated sec ASD | 3 days | Complication: 2 days post operatively, pt experienced device-related compression of the aortic root and AV node, leading to 2nd-degree heart block.  Management: Urgent surgical device removal & ASD closure. | ASO 24mm | ICE |
| 2015 | Embolism & Urgent Surgery | 11.7 | 34.3 | Isolated sec ASD | 1 day | Complication: Device was detected in the main pulmonary artery (MPA) on the 24-hour follow-up echo.  Management: Urgent surgical tricuspid valve repair, surgical device retrieval & ASD closure (performed successfully). | ASO 32mm | ICE |
